# Peroxisome deficiency impacts metabolites of lysine, lipid, and polyamine metabolism in *Saccharomyces cerevisiae*

**DOI:** 10.1007/s00418-025-02456-4

**Published:** 2026-01-29

**Authors:** Tjasa Kosir, Daniel Baptista Alves Malheiro, Lea Giørtz Johnsen, Hirak Das, Bettina Warscheid, Morten Danielsen, Ida J. van der Klei

**Affiliations:** 1 Molecular Cell Biology, Groningen Biomolecular Sciences and Biotechnology Institute (GBB), University of Groningen, PO Box 11103, 9300 CC Groningen, The Netherlands; 2 Cmbio (MS-Omics), Bygstubben 9, 2950 Vedbæk, Denmark; 3 Theodor Boveri-Institute, Biochemistry II, Biocenter, Faculty of Chemistry and Pharmacy, University of Würzburg, 97074 Würzburg, Germany

**Keywords:** Yeast, Peroxisomes, Metabolomics, Lysine, Carnitine, Glycerol-3-phosphate, Polyamines

## Abstract

Peroxisomes are eukaryotic organelles primarily known for their conserved roles in fatty acid β-oxidation and hydrogen peroxide detoxification. These organelles are also involved in a diverse range of other metabolic and non-metabolic functions. We recently compared the transcriptome and proteome of *Saccharomyces cerevisiae* wild-type and peroxisome-deficient (*pex3*) cells. This study uncovered the major processes and metabolic pathways that are influenced by peroxisomes. Here we performed a mass spectrometry-based analysis of intracellular metabolites of the same two strains. This led to the identification of 160 compounds, of which seven exhibited significant differences between wild-type and *pex3* cells (glycerol-3-phosphate, carnitine, pantothenate, acetyl-spermidine, propionyl-carnitine, and aminolevulinic acid). Notably, we observed elevated lysine levels in *pex3* cells, consistent with previous findings, which confirms the reliability and accuracy of our analytical approach. In addition, changes in carnitine compounds were measured, aligning with the proposed occurrence of a carnitine shuttle across the peroxisomal membrane. By integration of the current metabolomic data with the previously obtained transcriptomic and proteomic data, we provide a broader view of the metabolic impact of peroxisome deficiency. We show that, in addition to the well-known function of yeast peroxisomes in lipid and fatty acid degradation, these organelles are also involved in lipid synthesis. Furthermore, our study revealed that peroxisome deficiency affects polyamine homeostasis.

**Supplementary Information:**

The online version contains supplementary material available at 10.1007/s00418-025-02456-4.

## Introduction

Eukaryotic cells are organized into specialized, membrane-bound organelles, including peroxisomes, which house enzymes crucial for a variety of biochemical reactions and metabolic pathways. The most recognized and conserved metabolic function of peroxisomes is the β-oxidation of fatty acids (FA), along with the production and degradation of hydrogen peroxide (Imanaka 2019). In addition, peroxisomes play a role in a wide range of additional metabolic pathways as well as in non-metabolic processes. Some examples are the metabolism of plasmalogens in mammals (Honsho and Fujiki 2023) and pyrimidines in trypanosomatids (Tiwari and Dubey 2018), as well as polyamines in mammals, plants, and some yeast species (Zahedi et al. 2022). Non-metabolic pathways include a role of peroxisomes in stress response, aging, and cellular signaling (Dixit et al. 2010).

The yeast *Saccharomyces cerevisiae* is a widely used model organism. It shares many essential cellular processes and molecular mechanisms with higher eukaryotes, making it an ideal system for studying fundamental biological questions, including regarding the peroxisomal metabolome. *S. cerevisiae* peroxisomes have been implicated in several metabolic pathways. In addition to their well-established role in FA β-oxidation (Wang et al. 2024), the glyoxylate cycle (Kunze and Hartig 2013), and nicotinate/nicotinamide metabolism (Croft et al. 2020), peroxisomal enzymes are also part of biosynthetic pathways, such as lysine biosynthesis, because saccharopine dehydrogenase and Lys1 (Huh et al. 2003; Yofe et al. 2016) together with the Lys1 stabilizing protein Pls1, localize to peroxisomes (David et al. 2022). Notably, peroxisome deficiency leads to elevated intracellular concentrations of lysine in *S. cerevisiae* (Breitling et al. 2002; Gu et al. 2023), thereby reinforcing the association between lysine biosynthesis and peroxisomal function.

Recently, with the use of high-content fluorescence microscopy screens, a few new peroxisomal enzymes have been demonstrated to occur in *S. cerevisiae* peroxisomes. These include Bud16, which is involved in pyridoxal 5′-phosphate synthesis, and Hem14, a protoporphyrinogen oxidase which plays a role in heme biosynthesis. In addition to enzymes, proteins with regulatory roles, like the GID (glucose-induced degradation) complex subunits Gid2 and Gid7, which mediate glycolysis and gluconeogenesis, have been identified as part of the peroxisomal proteome (Yifrach et al. 2022). These discoveries suggest that additional, yet unknown, metabolic roles of yeast peroxisomes may exist.

Novel peroxisome-related pathways were also discovered upon comparing the transcriptome and whole-cell proteome of *S. cerevisiae* wild-type (WT) and a peroxisome-deficient mutant (*pex3*) (Kosir et al. 2025). This study led to the discovery of the dual localization of the pyruvate transporter complex Mpc1/3 at peroxisomes and mitochondria, demonstrating the role of yeast peroxisomes in pyruvate metabolism. Additionally, our study revealed that, beyond the changes in peroxisome-related processes, such as the enhancement of most β-oxidation enzymes, several non-peroxisomal transcripts and proteins also exhibited alterations.

We here set out to investigate changes in the intracellular metabolome using the same WT and peroxisome-deficient *pex3* strain as in our recent proteomics and transcriptomics study (Kosir et al. 2025). This revealed that the levels of several peroxisomal and non-peroxisomal metabolites were changed in the absence of peroxisomes. Integration of these data with the previously obtained transcriptomics and proteomics data revealed changes in known peroxisomal pathways (such as lysine and carnitine-related metabolism), as well as in compounds that were not yet linked to peroxisomes.

## Materials and methods

### Strains and growth conditions

The yeast strains used in this study are listed in Supplementary Table 3. Yeast cells were pre-cultivated on mineral medium containing glucose and shifted to medium containing acetate essentially as described previously (Kosir et al. 2025).

### Metabolomics

#### Sample preparation

To obtain the samples for the metabolomics analysis, cultures with a total OD_660_ of 16 were quenched in 25 ml of pure methanol (−80 °C), vortexed for 10 s, and filtered (Cytiva Supor 200). Metabolites were extracted in 5 ml of 40% (v/v) methanol, 40% (v/v) acetonitrile, and 20% (v/v) water by vortexing the filter with biomass for 30 s. Samples were dried under nitrogen flow and reconstituted by adding 500 µl of water. Samples were 10× diluted in mobile phase A for their respective liquid chromatography–mass spectrometry (LC–MS) method and filtered using a Costar^®^ Spin-X^®^ centrifuge tube 0.22 µm nylon membrane (Corning^®^) before analysis. Quality control (QC) samples were prepared by pooling small equal aliquots from each sample to create a representative average of the entire set.

#### LC–MS method and data processing

Modified protocols by Hsiao et al. (2018) and Doneanu et al. (2011) were used for polar and semi-polar metabolite profiling, respectively. The analysis was carried out in randomized order with an injection volume of 5 µl using an ultrahigh-performance liquid chromatography (UPLC) system (Vanquish, Thermo Fisher Scientific) coupled with a high-resolution quadrupole-orbitrap mass spectrometer (Q Exactive™ HF Hybrid Quadrupole-Orbitrap, Thermo Fisher Scientific) for the polar method. An Orbitrap Exploris 240™ (Thermo Fisher Scientific) was used for the semi-polar method. The ionization was achieved with an electrospray ionization interface operated in positive and negative ionization mode. Analysis was performed over an *m*/*z* range of 60–900 for the polar method at a mass resolution of 120,000 (at *m*/*z* 200), while the semi-polar method used an *m*/*z* range of 65–975 with polarity switching at a mass resolution of 60,000 (at *m*/*z* 200). A QC sample was analyzed by tandem mass spectrometry (MS/MS) using data-dependent acquisition at a resolution of 30,000 and normalized collision energy of 20, 40, and 60 eV for the identification of compounds. All solvents and additives were LC–MS grade (VWR Chemicals), and water was purified with a Direct-Q 3 ultraviolet (UV) water purification system with an LC-Pak polisher (Merck KGaA).

Data were processed using Compound Discoverer 3.3 (Thermo Fisher Scientific) and Skyline 22.2. Identification of compounds was performed on (i) level 1, with identification by retention times (compared against in-house authentic standards), accurate mass, and MS/MS spectra; (ii) level 2, with identification by retention times (compared against in-house authentic standards) and accurate mass; (iii) level 3a, with identification by accurate mass and MS/MS spectra; and (iv) level 4, with identification by accurate mass alone. A maximum mass deviation of 3 ppm was allowed. Results from the polar and semi-polar analyses were merged. Compound duplicates from levels 1 and 2 were removed. Polar results have a compound identifier starting with Y or SLY and were normalized to the mean of internal standards. Semi-polar results have a compound identifier starting with X or SL and were expressed as raw peak areas without normalization.

#### Data analysis

All metabolite profiling experiments were conducted with biological triplicates and technical duplicates. Two-tailed *t*-tests were used for comparisons between groups (*n* = 3) using average values of the technical duplicates. For the statistical analysis and data, plotting was performed with Excel. All compounds were given Kyoto Encyclopedia of Genes and Genomes (KEGG) identifiers and analyzed with the MetaboAnalyst (ver. 6.0) (Xia et al. 2009), Biocyc (Karp et al. 2019), and YeastPathways databases (Cherry 1998).

### Fluorescence microscopy

Wide-field fluorescence microscopy images of living cells were captured in growth medium at room temperature. Images were obtained using an Axioscope A1 microscope (Carl Zeiss), with a 100× 1.30 NA objective, Micro-Manager 1.4 software, and a digital camera (CoolSNAP HQ2; Photometrics). Green fluorescent protein (GFP) and mNeonGreen fluorescence was visualized using a 470/40 nm band-pass excitation filter and 495 nm dichromatic mirror. Image analysis was performed using ImageJ (Schneider et al. 2012).

## Results

### Overview of the metabolomics data

**Fig. 1:**
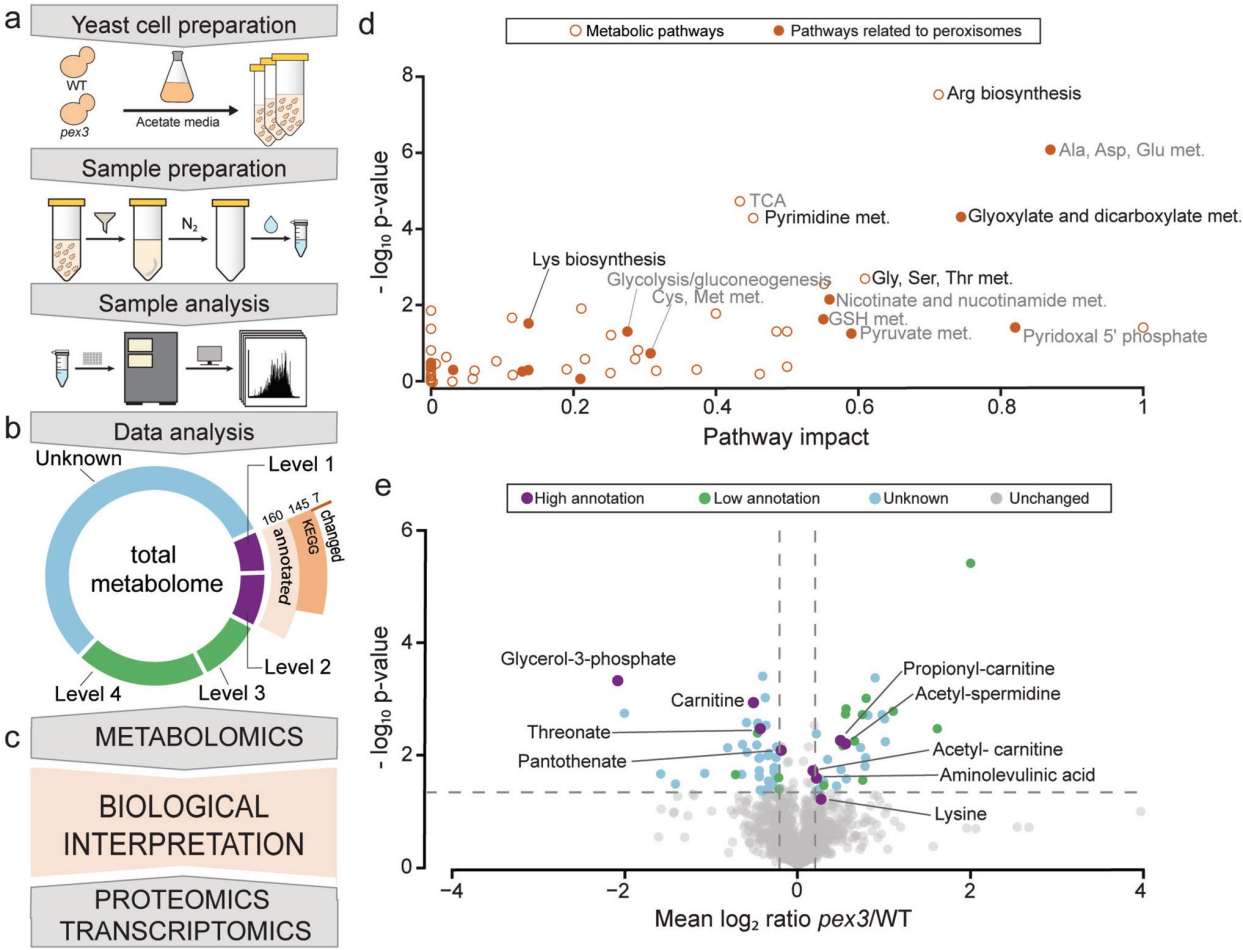
Metabolomics analysis of *S. cerevisiae* WT and *pex3* cells. **a** An overview of the experimental approach used to analyze global differences in the metabolome of WT and *pex3* cell growth on acetate media with a quantitative mass spectrometry approach for polar and semi-polar metabolite profiling. Experiments were performed in three independent biological and two technical replicates. **b** Overview of the detected metabolites, identified as follows: (i) level 1 (retention times, accurate mass, and MS/MS spectra, and compared against in-house authentic standards), level 2 (retention times and accurate mass and compared against in-house authentic standards), level 3 (accurate mass and MS/MS spectra), and level 4 (accurate mass). Metabolites identified at confidence levels 1 and 2 were compared against in-house authentic standards and are considered accurately annotated (160), with 145 recognized by KEGG and seven showing significant changes (∆). **c** The biological interpretation of the metabolomics data involved integrating the transcriptomics and proteomics data conducted using the same strains and cultivation conditions (Kosir et al. 2025). **d** Scatter plot of metabolic pathways of all highly annotated metabolites using MetaboAnalyst software. Pathway enrichment results were plotted against the corresponding −log_10_ *p*-value. Each point represents a metabolic pathway, with filled points indicating pathways that involve known peroxisomal proteins. **e** Global differences in metabolome in *pex3* versus WT cells. Mean log_2_ *pex3*/WT ratios were plotted against the corresponding −log_10_ *p*-value (two-sided *t*-test). Metabolites with a log_2_ ratio *pex3*/WT of < −0.2 or > 0.2 and *p* value threshold ≤ 0.05 (*n* = 3) were considered significantly changed. Each dot represents one metabolite.

To study the effect of the loss of peroxisomes on the intracellular metabolome, we compared *S. cerevisiae* WT and *pex3* cells using the same experimental conditions (batch cultures in mineral medium containing acetate as sole carbon source) as in our previous transcriptomics and proteomics comparison (Kosir et al. 2025). We used non-targeted profiling methods and an analytical approach for the detection of polar and semi-polar metabolites using mass spectrometry, allowing us to identify a wide range of compounds (Fig. 1a).

Among the samples of both strains, a total of 1822 compounds were detected at different levels of confidence. Of these, the annotation of 70 and 90 metabolites were recognized at level 1 (identified by retention times, accurate mass, and MS/MS spectra) and level 2 (identified by retention times and accurate mass), respectively. The remaining compounds were annotated with lower certainty (322), e.g., level 3 and 4, or unknown (1340) (Fig. 1b, Supplementary Table 1a). For further analysis, we confined our analysis to those with the highest annotation confidence levels (levels 1 and 2) (Supplementary Table 1b). To identify the metabolic pathways associated with these compounds, we performed a pathway analysis using MetaboAnalyst software (Xia et al. 2009). Of the 160 compounds, 145 were recognized as KEGG objects and subjected to pathway analysis. This analysis identified 58 metabolic pathways, primarily related to amino acid, energy, and cofactor metabolism. Notably, several of these pathways partially involve peroxisomal enzymes, including the glyoxylate cycle, lysine biosynthesis, and nicotinamide metabolism (Fig. 1d, Supplementary Table 1c).

Compounds with the mean log_2_ ratio *pex3*/WT of < −0.2 or > 0.2 and *p*-value threshold ≤ 0.05 (*n* = 3) were considered as having significant differences between *pex3* and WT cells. Out of the 1882 total metabolite entries, 311 met these criteria, with seven identified with high confidence. Of these, four were reduced in *pex3*—glycerol-3-phosphate (G3P), carnitine, threonate, and pantothenate—and three were enhanced in *pex3*—N8-acetyl-spermidine, propionyl-carnitine, and aminolevulinic acid. In addition, we observed borderline values for two compounds that are of interest for our study, namely lysine (log_2_ *pex3*/WT = 0.28, *p* = 0.07) and acetyl-carnitine (log_2_ *pex3*/WT = 0.18, *p* = 0.02) (Fig. 1e).

Differences in the intracellular metabolome of yeast cells result from changed activities or abundance of enzymes or transporters. To place the metabolomics results in the cell context, we integrated the metabolomics data with the outcome of the recently performed transcriptomics and proteomics comparison of *pex3* versus WT cells, which was obtained from the same experimental setup, such as acetate-grown cells harvested in the early exponential phase (Kosir et al. 2025) (Fig. 1c). Quantitative whole-cell proteomics analysis of WT and *pex3* cells identified 3967 proteins, of which 236 showed a significant change in abundance of at least 1.25-fold (75 decreased and 161 increased; *p* < 0.05, *n* = 3). Among these, 92 were annotated as peroxisomal proteins, with 15 decreased and 13 increased in abundance. Complementary transcriptomic analysis identified 5460 transcripts, including 123 peroxisomal genes. Of these, 3952 transcripts corresponded to proteins quantified in the proteomic dataset. A total of 91 peroxisomal genes were detected at both the transcript and protein levels, while an additional 32 peroxisomal transcripts were identified only by RNA sequencing. Overall, 102 transcripts were significantly regulated by at least 1.5-fold (25 upregulated and 77 downregulated; *p* < 0.05, *n* = 3) in *pex3* cells. (Supplementary Table 2).

### Intracellular lysine is higher in the absence of peroxisomes

**Fig. 2:**
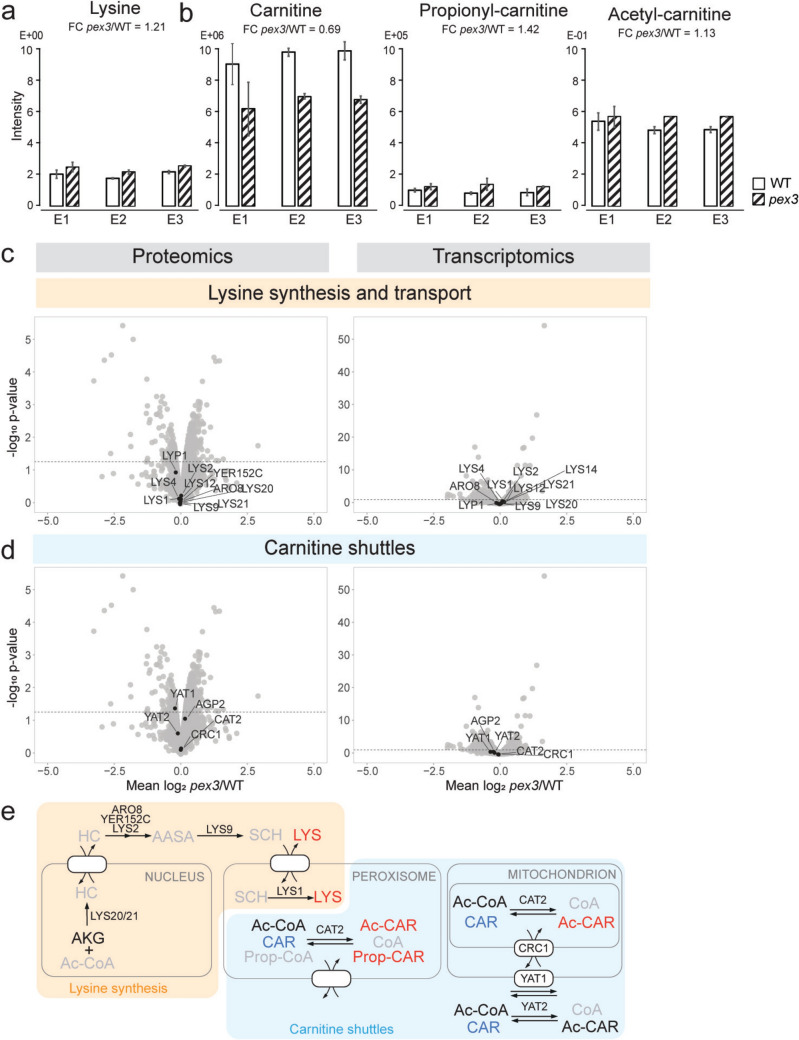
Peroxisome deficiency results in changes in lysine and carnitine-related compounds. **a** Intracellular levels of lysine in WT and *pex3* cells. Each bar represents the average of two measurements of the indicated metabolite in three biological replicates (E1, E2, and E3), from which the fold change (FC) was calculated. The bar represents the standard deviation. **b** Intracellular levels of carnitine, propionyl-carnitine, and acetyl-carnitine, as described in (**a**). **c**, **d** Volcano plots depicting the results of the quantitative proteomics (left) and transcriptomics (right) analyses of *pex3* versus WT cells (Kosir et al. 2025), highlighting the transcripts and proteins of lysine biosynthesis (**c**) and transport and carnitine shuttles (**d**). The horizontal line in the volcano plots indicates a *p*-value of 0.05. **e** Schematic representation of lysine biosynthesis pathway and carnitine-related pathways. Metabolites with a *p*-value of < 0.05 and a log_2_ FC of < −0.2 or > 0.2 are colored blue and red, respectively. Unchanged and undetected metabolites are depicted in black and gray (circles), respectively. Colored circles represent the transcripts (T, left) and proteins (P, right) that fulfill the *p*-value threshold of < 0.05. *AKG* α ketoglutarate, *Ac-CoA* acetyl-CoA, *HC* homocitrate, *AASA* aminoadipate semialdehyde, *SCH* saccharopine, *LYS* lysine, *CAR* carnitine, *Ac-CAR* acetyl-carnitine, *CoA* coenzyme A, *Prop-CAR* propionyl carnitine, *Prop-CoA* propionyl-CoA.

As shown in Fig. 2a, the intracellular levels of lysine are enhanced in *pex3* cells (*pex3*/WT = 1.21, *p* = 0.067), while intermediates of the lysine biosynthesis pathway were not detected. In *S. cerevisiae*, lysine is produced via the α-aminoadipate pathway (Xu et al. 2006), which involves enzymes in multiple subcellular compartments, including peroxisomes. Saccharopine dehydrogenase (Lys1) catalyzes the last step of lysine biosynthesis and is peroxisomal (Huh et al. 2003; Yofe et al. 2016). In addition, a few other lysine biosynthetic enzymes (Lys4 and Lys2, Lys9) may be peroxisomal as well because they contain predicted peroxisomal targeting signals (PTS1 and PTS2, respectively) (Breitling et al. 2002) (Fig. 2e). The levels of transcripts and proteins involved in lysine biosynthesis, including the lysine biosynthesis transcription factor Lys14, were unchanged (Fig. 2c). The recently discovered peroxisomal lysine synthesis regulator Pls1 (David et al. 2022) was not detected in the proteomics analysis (Kosir et al. 2025). Next to the peroxisomal localization of Lys1, the elevated lysine levels in *pex3* cells underscore the involvement of peroxisomes in lysine biosynthesis.

### The absence of peroxisomes influences the intracellular concentrations of carnitine compounds

Our metabolomics data show that carnitine levels are reduced (*pex3*/WT = 0.69, *p* = 0.001), while propionyl-carnitine is increased in *pex3* cells (*pex3*/WT = 1.42, *p* = 0.006). A slight increase was also detected for acetyl-carnitine (*pex3*/WT = 1.13, *p* = 0.021) (Fig. 2b). No significant change was measured for acetyl-CoA (Supplementary Fig. 1a), while free CoA was not detected.

Carnitine is a key metabolite in energy metabolism, functioning as a shuttling molecule of acyl residues across cellular membranes. Acetyl-carnitine is shuttled across the peroxisomal membrane during β-oxidation of FA (Wang et al. 2024), generated from acetyl-CoA in the peroxisomal matrix by carnitine acetyl-CoA transferase, Cat2 (Elgersma et al. 1995). Moreover, acetyl-carnitine is imported into peroxisomes during the growth of cells on acetate, where it is the substrate of the peroxisomal glyoxylate cycle enzyme citrate synthase (Cit2) (Kunze et al. 2006). A peroxisomal transporter of carnitine compounds has not yet been identified (Fig. 2e). Cat2 and Cit2 were unchanged at the transcriptomics and proteomics level, as well as other carnitine-related proteins. These include carnitine acetyl-CoA transferase Yat1 and Yat2 (Schmalix and Bandlow 1993; Elgersma et al. 1995; Swiegers et al. 2001) and the carnitine transporters Crc1 and Agp2 (van Roermund 1999; Palmieri et al. 1999) (Fig. 2d) (Kosir et al. 2025). Propionyl-carnitine and propionyl-CoA molecules likely rely on the same set of proteins for their transport or metabolism.

Other metabolic pathways that lead to the production of propionyl-CoA are the degradation of threonine, methionine, valine, and isoleucine. Propionyl-CoA can also be produced from propionate via acetyl-CoA synthetase (Frenkel and Kitchens 1977; Patel and Walt 1987), which is also a byproduct of amino acid degradation. We were able to detect all amino acids, but except for lysine, none of them demonstrated a changed intracellular concentration in the absence of peroxisomes (Supplementary Fig. 1b).

Carnitine and carnitine shuttles are also involved in stress protection; for example, peroxisomal and mitochondrial Cat2 contributes to the response to oxidative stress (Franken et al. 2008). The absence of peroxisomes could lead to changes in oxidative stress and in this way influence carnitine levels. However, transcripts and proteins of enzymes involved in producing reactive oxygen species or antioxidant defense remained largely unchanged in the absence of peroxisomes (Kosir et al. 2025), indicating that changes in the carnitine compounds in *pex3* cells are not due to oxidative stress.

### Reduced glycerol-3-phosphate levels suggest changes in glycerolipid metabolism in *pex3* cells

**Fig. 3:**
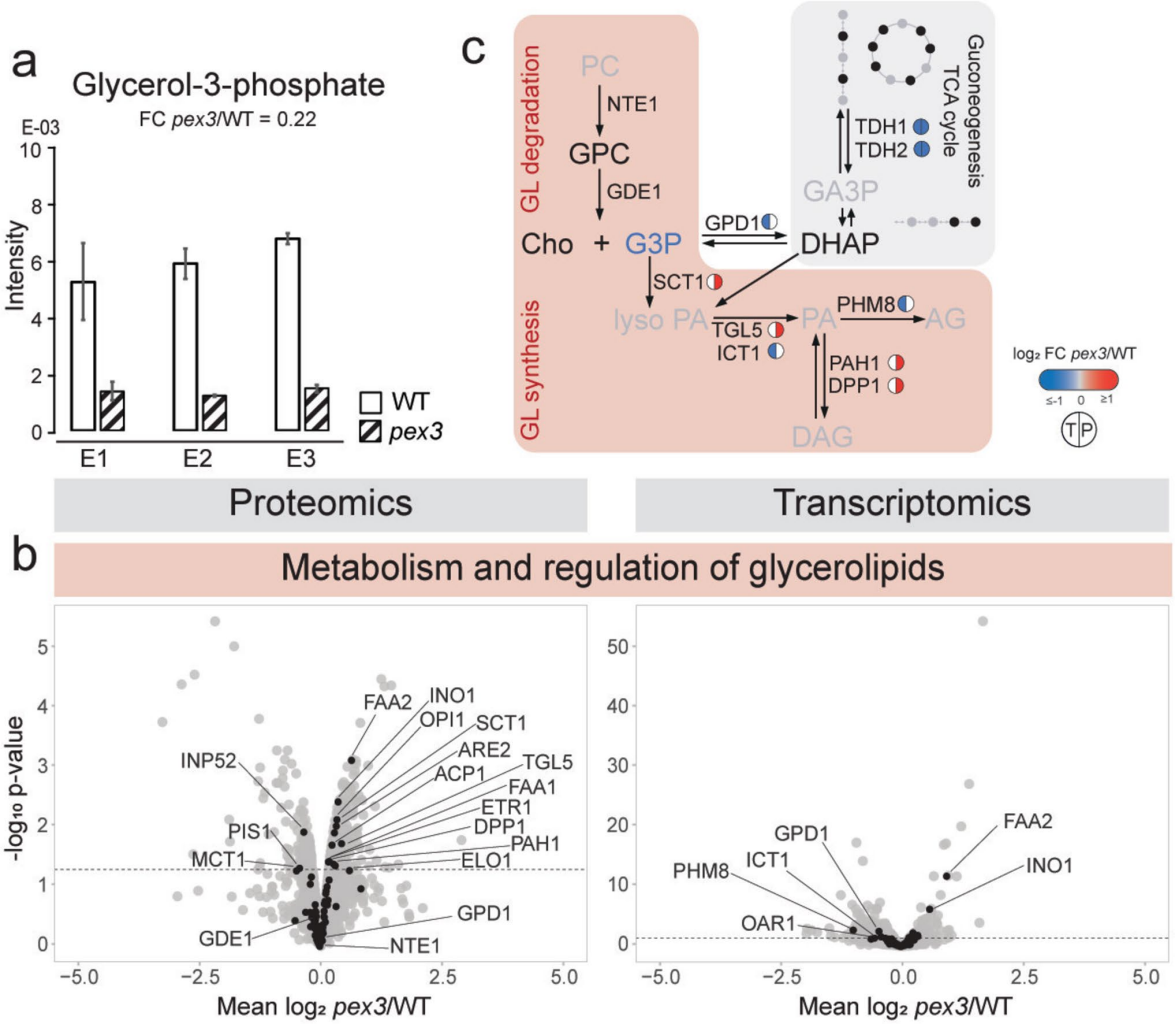
Effects of peroxisomal deficiency on pathways involved in glycerolipid metabolism. **a** Intracellular levels of glycerol-3-phosphate (G3P). **b** Volcano plots highlight the transcripts (right) and proteins (left) of the metabolism and regulation of glycerolipids. **c** Schematic representation of glycerolipid (GL) synthesis and degradation processes, as well as TCA cycle and gluconeogenesis. For a detailed description of the figure, see legend of Fig. 2. Circles represent the transcripts (T, left) and proteins (P, right). Transcripts/proteins that fulfill the *p*-value threshold of < 0.05 are colored. Unchanged and undetected transcripts/proteins are depicted in gray and white, respectively, or without circles. *PC* phosphatidyl choline, *GPC* glycerophosphocholine, *Cho* choline, *G3P* glycerol-3-phosphate, *DHAP* dihydroxyacetone phosphate, *PA* phosphatidic acid, *D(AG)* (di)acylglycerol, *GA3P* glyceraldehyde-3-phosphate

Our metabolomics data revealed that G3P levels were reduced in *pex3* cells relative to the WT control (Fig. 3a). G3P can be a product of gluconeogenesis by NAD-dependent glycerol-3-phosphate dehydrogenases, Gpd1 and Gpd2 (Fig. 3c). Gpd1 is a peroxisomal enzyme (Effelsberg et al. 2015) and, like Gpd2 (Eriksson et al. 1995), converts dihydroxyacetone phosphate (DHAP) into G3P. DHAP is a metabolite of gluconeogenesis and glycolysis. The relation between the peroxisomes and gluconeogenesis was also shown by the peroxisomal localization of GID complex components Gid2 and Gid7 in cells grown on non-fermentable carbon sources (Yifrach et al. 2022).

Several metabolites of the gluconeogenesis pathway and the tricarboxylic acid (TCA) cycle were detected but unchanged (Supplementary Fig. 1c, d). This is also the case for a majority of enzyme transcripts and proteins, including protein levels of Gpd1 and GID complex components (Supplementary Fig. 2a, b). Exceptions were glyceraldehyde-3-phosphate dehydrogenases TDH1 and TDH2 (Fig. 3c, Supplementary Fig. 2a), which were lower at the transcript and protein level, potentially leading to the production of lower amounts of glyceraldehyde-3-phosphate. This metabolite was not detected in metabolomics. Since WT and *pex3* cells have the same growth rate and acetate consumption, we can assume that the availability and/or conversions related to primary metabolites do not contribute to changes in the G3P.

G3P is also a key metabolite in phospholipid synthesis. Disruptions or imbalances in phospholipid synthesis can lead to changes in G3P levels (Fig. 3c). Opi1, a protein with higher abundance in *pex3* cells, is a transcriptional regulator in this process. Since Opi1 regulation is mediated by protein localization, we do not know how changes in protein levels affect its regulatory function. However, higher protein and transcript abundance of Ino1 in *pex3*, a protein directly regulated by Opi1 (Ravi et al. 2019), suggests that Opi1 did not repress the glycerophospholipid synthesis genes. Ino1, an inositol-3-phosphate synthase, is involved in the synthesis of inositol phosphates and inositol-containing phospholipids. Two other proteins related to inositol phospholipids, polyphosphatidyl-inositol phosphatase Inp52 and phosphatidyl-inositol synthase Pis1, were both reduced in *pex3* cells (Fig. 3b). This suggests that inositol-containing lipids might be changed in the absence of peroxisomes.

A few other proteins involved in the synthesis and regulation of glycerolipids were changed as well. As shown in Fig. 3b, this includes Sct1, an endoplasmic reticulum (ER) protein with G3P/DHAP dual substrate acyltransferase activity involved in the production of lysophosphatidic acid (LPA) (Minskoff et al. 1994; Matsushita and Nikawa 1995; Zheng and Zou 2001; Bratschi et al. 2009), and Tgl5, a lipid droplet (Athenstaedt and Daum 2005) bifunctional enzyme with triacylglycerol lipase and LPA acyltransferase activity, producing free FA and phosphatidic acid (PA), respectively (Rajakumari and Daum 2010). Sct1 and Tgl5 had a higher protein abundance in the *pex3* cells, as well as Pah1 (PA phosphatase) and Dpp1 (diacylglycerol pyrophosphate phosphatase) (FC = 1.22). Changes in lipid synthesis are also suggested by the increase in transcript levels of glycerolipid precursor enzymes, such as the peroxisomal medium chain fatty acyl-CoA synthetase Faa2 and FA elongase Elo3, and a decrease in LPA phosphatase Phm8, LPA acyltransferase Ict1, and acyl-carrier-protein Oar1 (Kosir et al. 2025). Altogether, these results highlight the considerable influence of peroxisome deficiency on the glycerolipid biosynthesis pathways.

### Absence of peroxisomes affects polyamine homeostasis

**Fig. 4:**
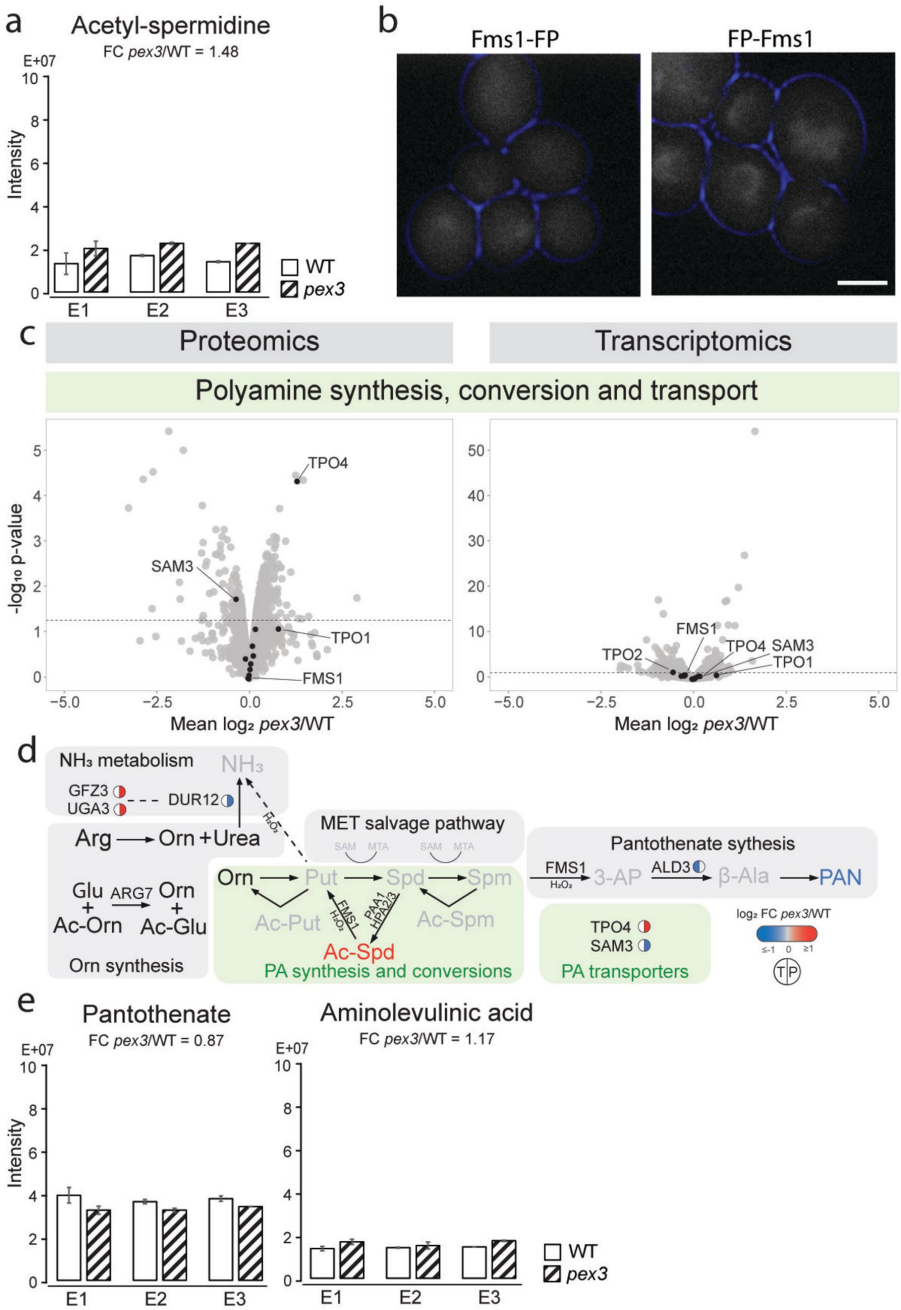
Polyamine and related pathways respond to peroxisome deficiency. **a** Intracellular levels of acetyl-spermidine. **b** Fluorescence microscopy analysis of acetate-grown *S. cerevisiae* expressing Fms1 with the fluorescent protein (FP) NeonGreen at the C-terminus under the control of the endogenous promoter or GFP at the N-terminus under the control of the *NOP1* promoter. The cell contour is shown in blue. Scale bar: 2.5 μm. **c** Volcano plots highlight the transcripts (right) and proteins (left) of polyamine synthesis, conversion, and transport. **d** Schematic representation of polyamine metabolism and related pathways. **e** Intracellular levels of pantothenate and aminolevulinic acid. For a detailed description of this figure, see legend of Fig. 2. Circles represent the transcripts (T, left) and proteins (P, right). Transcripts/proteins that fulfill the *p*-value threshold of < 0.05 are colored. Unchanged and undetected transcripts/proteins are depicted in gray and white, respectively, or without circles. *Arg* arginine, *Orn* ornithine, *Put* putrescine, *Ac-Put* acetyl-putrescine, *Spd* spermidine, *Ac-Spd* acetyl-spermidine, *Spm* spermine, *Ac-Spm* acetyl-spermine, *SAM* S-adenosylmethionine, *MTA* 5′-methylthioadenosine, *3-AP* 3-aminopropanal, *β-Ala* β-alanine, *PAN* pantothenate

We detected an increased level of acetyl-spermidine in *pex3* cells (Fig. 4a). Other polyamines, or their acetylated versions, remained unchanged or were not detected, along with molecules involved in polyamine biosynthesis, such as S-adenosylmethionine (SAM), S-methyl-5′-thioadenosine, and ornithine (Subhi et al. 2003) (Supplementary Fig. 1f). Acetylation of polyamines is a protective mechanism cells use to mitigate polyamine toxicity, suggesting that, in the absence of peroxisomes, cells may produce higher amounts of polyamine spermidine.

Peroxisomal involvement in polyamine metabolism is related to their retroconversions, including acetylation and oxidation. Peroxisomal polyamine oxidases have been found in several species, including humans (HsPAOX), plants (AtPAO2, AtPAO3, AtPAO4), and the yeast *Candida boidinii*. *S. cerevisiae* has one polyamine oxidase, Fms1, which catalyzes the oxidation of acetylated and non-acetylated polyamines (reviewed in Salvi and Tavladoraki 2020). Fluorescence microscopy analysis of *S. cerevisiae* Fms1 tagged with a fluorescent protein (FP) at the C- or N-terminus resulted in faint cytosolic signals when cells were grown on acetate media, meaning that Fms1 is not peroxisomal at the experimental conditions used (Fig. 4b).

In addition, concentrations of intracellular polyamines can be controlled through their synthesis and influx/efflux in/out of the cell. As shown in Fig. 4c, the increased intracellular acetyl-spermidine levels were not due to changes in the transcripts or protein abundance of polyamine biosynthesis enzymes, including Fms1, or enzymes involved in the pathways that produce their substrates. However, we observed differences in two transporters, Sam3 and Tpo4, both of which can transport polyamines, further indicating that polyamine homeostasis is affected in *pex3* cells.

High protein levels of Tpo4, a polyamine exporting transporter, and low levels of Sam3, a polyamine uptake transporter, suggest that *pex3* cells regulate intracellular polyamine concentrations by polyamine export. However, deletion of *TPO4* in WT or *pex3* mutants does not result in a reduced growth phenotype (Kosir et al. 2025), suggesting that the loss of polyamine export via *Tpo4* does not lead to cell toxicity.

An alternative pathway in polyamine metabolism involves their use as a nitrogen source. This process is well understood in plants, where putrescine is converted to GABA, resulting in ammonia production (reviewed in Tyagi et al. 2023). However, the role of polyamines as nitrogen sources is less well studied in other eukaryotes. *C. boidinii* can grow on spermidine or putrescine as its sole nitrogen source (Haywood and Large 1985). In our study of *S. cerevisiae pex3* cells, several genes related to nitrogen metabolism were altered, including urea amidolyase (Dur12) and the transcription factors Gzf3 and Uga3 (Supplementary Table 1, Fig. 4d). This suggests that polyamines may also play a role in nitrogen metabolism in *S. cerevisiae*, and that this pathway is altered in the absence of peroxisomes.

Polyamines are also involved in the cell response to stress. Exporting polyamines prevents their oxidation and formation of hydrogen peroxide (Krüger et al. 2013). Another strategy is the uptake of lysine and its conversion to the alternative polyamine, cadaverine, thus channeling the lysine biosynthesis consuming NADPH towards glutathione production and increasing oxidant tolerance (Olin-Sandoval et al. 2019). In metabolomics data, glutathione and cadaverine were not changed and detected, respectively, meaning that this process most likely does not occur in peroxisome-deficient yeast cells.

### Miscellaneous compounds

Our metabolomics study led to the discovery of a few other compounds that are marginally related to the already described topics or did not correlate with the transcriptomics and proteomics data. One of them is pantothenate, which can be seen as part of the polyamine synthesis pathway (Fig. 4d). Pantothenate, which is slightly decreased in *pex3* cells (Fig. 4e), is produced when polyamines are used as substrates. Polyamine oxidase, Fms1, catalyzes the conversion of spermine to 3-aminopropanal, which is a rate-limiting reaction in the production of pantothenate (White et al. 2001). In addition, pantothenate was supplemented in the media. Fen2, a plasma membrane H+-pantothenate symporter (Stolz and Sauer 1999), was not detected at the protein level, suggesting that changes in pantothenate are not due to differences in pantothenate uptake, but the result of unbalanced intracellular polyamine concentrations.

Aminolevulinic acid, a key component in heme biosynthesis, was decreased in *pex3* cells (Fig. 4e). Heme biosynthesis enzymes are localized to mitochondria and the cytosol (Hoffman et al. 2003). It was recently shown that protoporphyrinogen oxidase Hem14 is a peroxisomal enzyme (Yifrach et al. 2022). However, we did not see any differences in the heme enzymes at the transcript and protein levels in *pex3* cells (Supplementary Fig. 2c).

Lastly, peroxisome-deficient cells contain lower content of threonic acid (Fig. 1c). Threonic acid is a sugar acid derivative of threose, a metabolite of ascorbic acid. *S. cerevisiae* is unable to synthesize ascorbic acid and lacks enzymes involved in the synthesis of this vitamin. Therefore, it is likely that the compound we detected is not threonic acid but a yet-unknown compound that has high structural similarity and the same accurate mass as threonic acid.

## Discussion

To investigate the effect of peroxisome deficiency on the yeast intracellular metabolome, we compared the metabolomes of acetate-grown *S. cerevisiae* WT and peroxisome-deficient (*pex3*) cells using an untargeted mass spectrometry approach. We identified a diverse array of compounds, with seven compounds exhibiting significant differences in the absence of peroxisomes. This included decreased levels of glycerol-3-phosphate, carnitine, threonate, and pantothenate, and increased levels of N8-acetylspermidine, propionyl-carnitine, and aminolevulinic acid. In addition, lysine and acetyl-carnitine, although not significantly altered, displayed consistent trends toward higher and lower abundance in *pex3* cells, respectively.

These metabolic shifts prompted us to investigate which pathways could explain the observed differences. Therefore, we reanalyzed the proteomics and transcriptomics data of the same strains grown under the same conditions. In summary, proteomic analysis of *pex3* cells revealed reduced abundance of peroxisomal membrane proteins despite unchanged transcript levels. Most matrix proteins remained unchanged, except for the enzymes of the β-oxidation pathway. Loss of *PEX3* also impacted pathways linked to mitochondrial protein import, translation, and respiration, as well as the pentose phosphate pathway, nitrogen metabolism, glycolysis, and gluconeogenesis (Kosir et al. 2025).

Consistent with the peroxisomal matrix genes and proteins, Lys1 and Cat2 showed no differences in transcript or protein abundance. Nevertheless, the absence of peroxisomes still resulted in altered lysine and carnitine levels, underscoring the fact that metabolic changes can arise independently of changes in protein expression.

Enhanced intracellular lysine concentrations are consistent with previous studies on peroxisome-deficient yeast mutants. Analysis of microarray data obtained from cells of the peroxisome-deficient *S. cerevisiae pex12* strain (Hughes et al. 2000) showed that genes encoding enzymes of the lysine biosynthesis pathway are upregulated. It was proposed that enhanced intracellular lysine levels would occur due to the activation of the transcriptional activator Lys14 by aminoadipate semialdehyde. Lys2 is an aminoadipate semialdehyde-producing enzyme with a predicted peroxisomal localization, which in *pex12* cells would mislocalize to the cytosol, leading to cytosolic production of aminoadipate semialdehyde-activating Lys14, and consequently expression of genes of the lysine biosynthesis pathway (Feller et al. 1994; Breitling et al. 2002). This mechanism does not appear to underlie the lysine increase in *pex3* cells, as we observed no alterations in the expression or abundance of lysine biosynthetic enzymes.

Another lysine-related study was performed in the fission yeast *Schizosaccharomyces japonicus*. Intracellular lysine levels were found to be fourfold higher in a mutant lacking the PTS1 receptor Pex5 (*pex5*) (Gu et al. 2023). Similar to *S. cerevisiae* Lys1, saccharopine dehydrogenase in *S. japonicus,* Lys3, is a peroxisomal protein containing a PTS1. Hence, in cells lacking Pex5, import of Lys3 is blocked, leading to its cytosolic mislocalization. It was suggested that higher intracellular lysine concentrations in *S. japonicus pex5* cells are due to the availability of NAD+, which might also be the case of *S. cerevisiae pex3* cells*.*

*S. cerevisiae* and *S. japonicus* saccharopine dehydrogenases require NAD+ as a cofactor. In these yeast species, the peroxisomal reoxidation of NADH to NAD+ depends on glycerol-3-phosphate dehydrogenase (Al-Saryi et al. 2017; Gu et al. 2023). Additionally, *S. cerevisiae* possesses the malate/oxaloacetate NAD+/NADH shuttle mediated by Mdh2 and Mdh3 (Al-Saryi et al. 2017). When peroxisomal compartmentalization is absent, these processes become non-limiting for lysine biosynthesis, as the cytosolic NAD+ pool can support lysine production, leading to higher intracellular lysine concentrations. In addition, this shift could potentially reduce the formation of G3P by glycerol-3-phosphate dehydrogenase, which could also contribute to the lower levels of G3P in *S. cerevisiae pex3* cells. However, *S. cerevisiae* exhibits only minor changes in internal lysine levels compared to *S. japonicus*. This discrepancy might result from differences in the negative feedback mechanisms regulating the lysine biosynthesis pathway.

Free lysine inhibits the first step of the lysine biosynthesis pathway, which is catalyzed by homocitrate synthase. *S. japonicus* homocitrate synthase (SjLys4) localizes to mitochondria (Gu et al. 2023), while its homologs in *S. cerevisiae* (ScLys20/21) are distributed throughout the cytosol and/or the nucleus (Chen et al. 1997; Feller et al. 1999). Compartmentalization may shield *S. japonicus* Lys4 from such inhibition, allowing high levels of lysine production. In contrast, in *S. cerevisiae*, free lysine partially inhibits ScLys20/21, leading to only a moderate increase in intracellular lysine.

In *S. cerevisiae pex3*, we observed no changes in the protein levels of lysine-related enzymes (Kosir et al. 2025), similar to that observed in *S. japonicus pex5* cells (Gu et al. 2023), indicating that the increase in intracellular lysine in both yeast species is not due to increased enzyme levels, but most likely related to substrate or cofactor availability.

The higher intracellular lysine levels observed in the *S. cerevisiae pex3* cells support the reliability of our method for measuring intracellular metabolites, as it produced results consistent with those seen in peroxisome mutants of other yeasts (Gu et al. 2023). Moreover, deletions of peroxisome-related genes were associated with alterations in amino acid levels in a previous study (Nötzel et al. 2016); however, unlike our results, *PEX3* deletion in the study of Yofe et al. (2017) did not affect lysine abundance. This discrepancy may arise from differences in growth conditions, as those studies used a medium containing glucose and all amino acids, whereas we employed a medium containing acetate supplemented only with the amino acids required for growth.

In addition to lysine, changes in carnitine compounds align with the presumed presence of a carnitine shuttle in the peroxisomal membrane, which is required in processes like FA β-oxidation.

Unlike higher eukaryotes, FA β-oxidation is confined to peroxisomes in *S. cerevisiae* (Wang et al. 2024). We previously showed that β-oxidation enzymes were significantly enhanced at both the transcript and protein level in *pex3* cells, except fatty-acyl coenzyme A oxidase (Pox1), which was reduced at the protein level in *pex3* cells (Kosir et al. 2025). This suggests that in the absence of peroxisomes, FA degradation may be reduced, despite the enhanced levels of several β-oxidation enzymes, potentially leading to changes in auxiliary compounds like carnitine.

We cannot conclusively attribute the changes in carnitine compounds to alterations in β-oxidation, as the methods used did not allow detection of FA. Small to no changes in acetyl-carnitine and acetyl-CoA levels, respectively, suggest that the metabolic flux remains stable, indicating that processes related to energy production and FA oxidation are intact. This is expected, as cells of both WT and *pex3* strains exhibit similar growth rates and acetate consumption (Kosir et al. 2025). However, we did observe elevated levels of propionyl-carnitine in *pex3* cells, another product of the β-oxidation pathway.

Propionyl-carnitine is formed when a propionyl group is transferred from propionyl-CoA to carnitine. In humans, carnitine acetyltransferase HsCat1 accepts various short-chain acyl-CoAs as substrates, including propionyl-CoA (Violante et al. 2013). This is probably also the case for the *S. cerevisiae* acetyltransferase Cat2. Propionyl-CoA is a metabolite in the catabolism of many compounds, including FA. Transfer of the propionyl group may help to maintain the acyl-CoA/CoA ratio and stimulate or inhibit FA metabolism (Siliprandi et al. 1991; Wang et al. 2018). The idea that peroxisomes play a regulatory role by controlling the availability of CoA was also suggested for *S. cerevisiae* (Van Broekhoven et al. 1981).

Carnitine compounds present an intriguing aspect of *S. cerevisiae* biology. In contrast to most eukaryotes, *S. cerevisiae* is unable to synthesize carnitine (van Roermund et al. 1995; Swiegers et al. 2001) and relies on carnitine uptake from the extracellular environment. Carnitine, as a limited intracellular resource, can be considered not only an auxiliary but also a regulatory compound. Exploring the regulatory role of carnitine-related molecules in peroxisome biology, particularly propionyl-carnitine influence on lipid synthesis, turnover, and degradation, would be of significant interest.

Another compound linked to the lipid metabolism is G3P. Lipid synthesis is regulated through phosphatidylcholine (PC) and Opi1. PC is a major lipid of eukaryotic cells, regulating the synthesis of other phospholipids. In *S. cerevisiae*, the activity of Nte1 controls the PC content of the ER, which impacts Opi1, the main transcriptional regulator of phospholipid synthesis (Fernández-Murray et al. 2009). Opi1 is associated with the ER membrane via binding to phosphatidic acid (PA). When PA levels are reduced, Opi1 dissociates from the ER and enters the nucleus, where it functions as a transcriptional repressor of phospholipid biosynthesis genes (Santiago and Mamoun 2003; Gaspar et al. 2017). We were not able to detect intracellular concentrations of lyso-PA or PA; however, Opi1 and a few enzymes related to lipid synthesis had a higher protein abundance in *pex3* cells. Together these emphasize the considerable influence of peroxisome deficiency on the glycerolipid biosynthesis pathways.

We observed significant changes in acetylated spermidine levels, indicating alterations in polyamine homeostasis in *pex3* cells. Polyamines are ubiquitous molecules that occur in all living organisms. The most common polyamines in fungi are putrescine, spermidine, and spermine. Since intracellular excess is cytotoxic, polyamine homeostasis is tightly regulated throughout synthesis, degradation, and retroconversion, as well as with uptake and excretion (Rocha and Wilson 2019).

While polyamine metabolism is associated with peroxisomes in other species, our findings suggest that this might also be the case for yeast. Even though extensive studies have been performed in *S. cerevisiae* (including organelle proteomics and high-throughput FM), peroxisomal localization of enzymes involved in polyamine metabolism was not established. Indeed, our fluorescence microscopy analysis of the localization of the polyamine oxidase Fms1 did not reveal a peroxisomal localization.

Perturbation of polyamine homeostasis in the absence of peroxisomes is also suggested by the observed changes in the protein abundance of polyamine transporters. Several plasma membrane transporters have been identified that are capable of transporting polyamines. For uptake, these include the SAM transporter Sam3, the urea transporter Dur3, the general amino acid transporter Gap1, the amino acid permease Agp2 (Rocha and Wilson 2019), and the high-affinity polyamine transporter Hal1 (Vindu et al. 2021). *S. cerevisiae* has four *TPO (1–4)* genes encoding polyamine transporters that mediate polyamine efflux, with Tpo4 specifically recognizing spermine, putrescine, and spermidine (Rocha and Wilson 2019). Two polyamine transporters showed changes in the protein abundance in *pex3* cells, Tpo4 and Sam3.

Reduced cell growth by blockage of polyamine export would suggest polyamine accumulation and toxicity. However, this was not the case for WT or *pex3* cells in the absence of Tpo4 (Kosir et al. 2025). We cannot exclude the possibility that other polyamine transporters, such as Tpo1, Tpo2, or Tpo3, may compensate for the absence of Tpo4 by taking over the polyamine efflux function or that other polyamine detoxification or metabolic processes took place.

Studying polyamines represents a challenge, since polyamines are involved in multiple pathways and have many regulatory mechanisms. Nevertheless, our data suggest a connection between peroxisomes and polyamines in *S. cerevisiae*.

Integrating metabolomics with previously conducted transcriptomics and proteomics analyses of *pex3* and WT cells enabled the identification of pathways directly or indirectly linked to peroxisomes. However, untargeted metabolomics poses challenges in compound annotation. Further investigation would entail analyzing samples with standards specific to the pathways of interest, confirming the changes in metabolites that lack confident annotation. With this work, we show that the approach is sufficient for the analysis of the intracellular metabolome of the peroxisome-deficient yeast cells.

## Supplementary Information

Below is the link to the electronic supplementary material.

Supplementary file1 (DOCX 2129 KB)

Supplementary file2 (XLSX 691 KB)

Supplementary file3 (XLSX 1164 KB)

## Data Availability

Metabolomics data have been deposited to the EMBL-EBI MetaboLights database (Yurekten et al., 2024), with the identifier MTBLS11793 ( https://www.ebi.ac.uk/metabolights/MTBLS11793).
