# Supplementary material for: Peroxisome deficiency impacts metabolites of lysine, lipid, and polyamine metabolism in Saccharomyces cerevisiae
Source: Histochem Cell Biol. 2026 Jan 29;164(1):6. doi: 10.1007/s00418-025-02456-4 (PMC12855314; doi:10.1007/s00418-025-02456-4)
Supplement: Supplementary file 1 — Supplementary file1 (DOCX 2129 KB) [file 418_2025_2456_MOESM1_ESM.docx]

**Peroxisome deficiency impacts metabolites of lysine, lipid and polyamine metabolism in *Saccharomyces cerevisiae***

Journal: Histochemistry and Cell Biology

Tjasa Kosir^a^, Daniel Baptista Alves Malheiro^b^, Lea Giørtz Johnsen^b^, Hirak Das^c^, Bettina Warscheid^c^, Morten Danielsen^b^, Ida J. van der Klei^a^

a Molecular Cell Biology, Groningen Biomolecular Sciences and Biotechnology Institute (GBB), University of Groningen, PO Box 11103, 9300 CC Groningen, The Netherlands

b Cmbio (MS-Omics), Bygstubben 9, 2950 Vedbæk, Denmark

c Theodor Boveri-Institute, Biochemistry II, Faculty of Chemistry and Pharmacy, University of Würzburg, 97074 Würzburg, Germany

Corresponding author: i.j.van.der.klei@rug.nl

# Supplementary information

## Supplementary figures


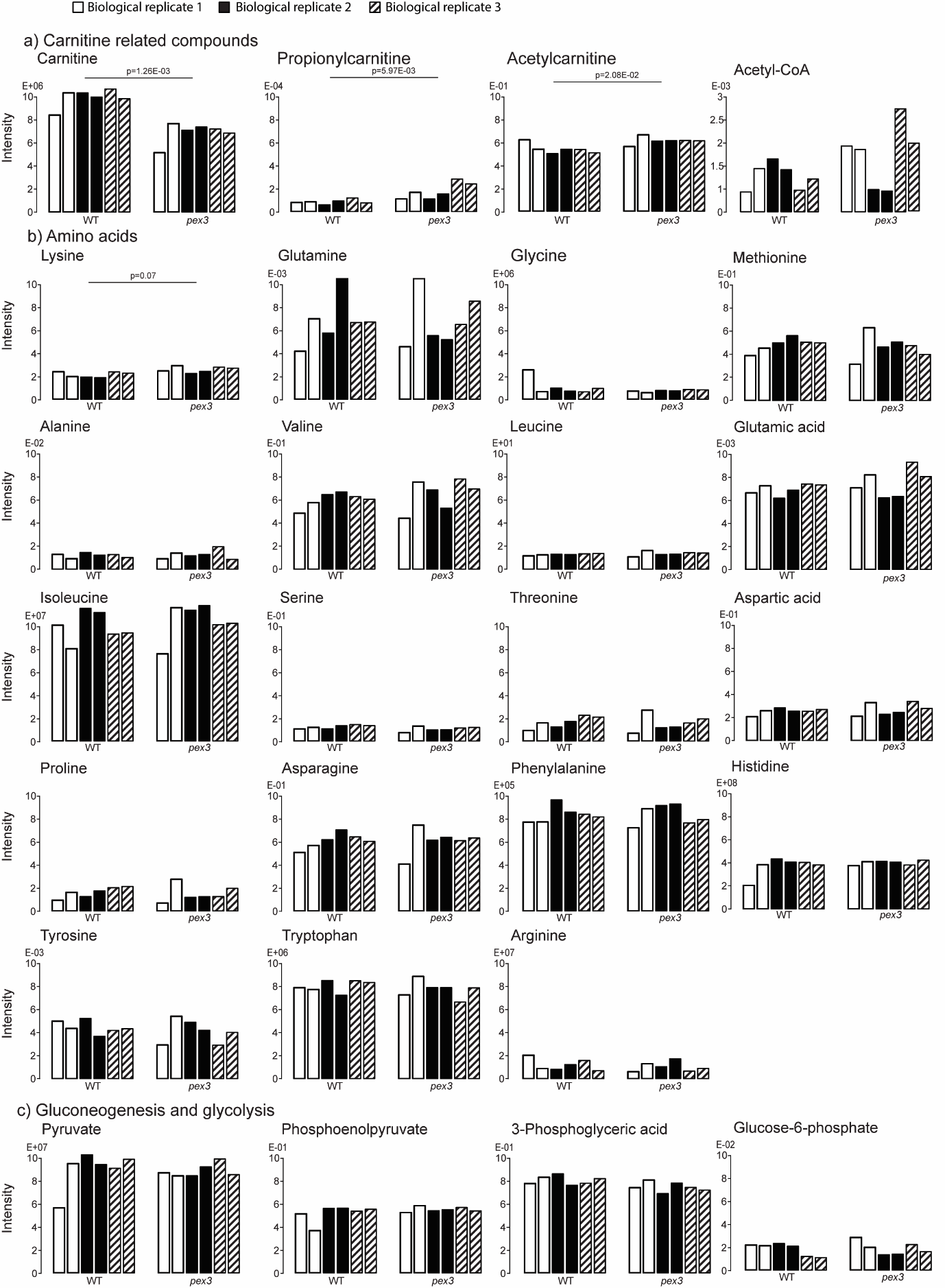


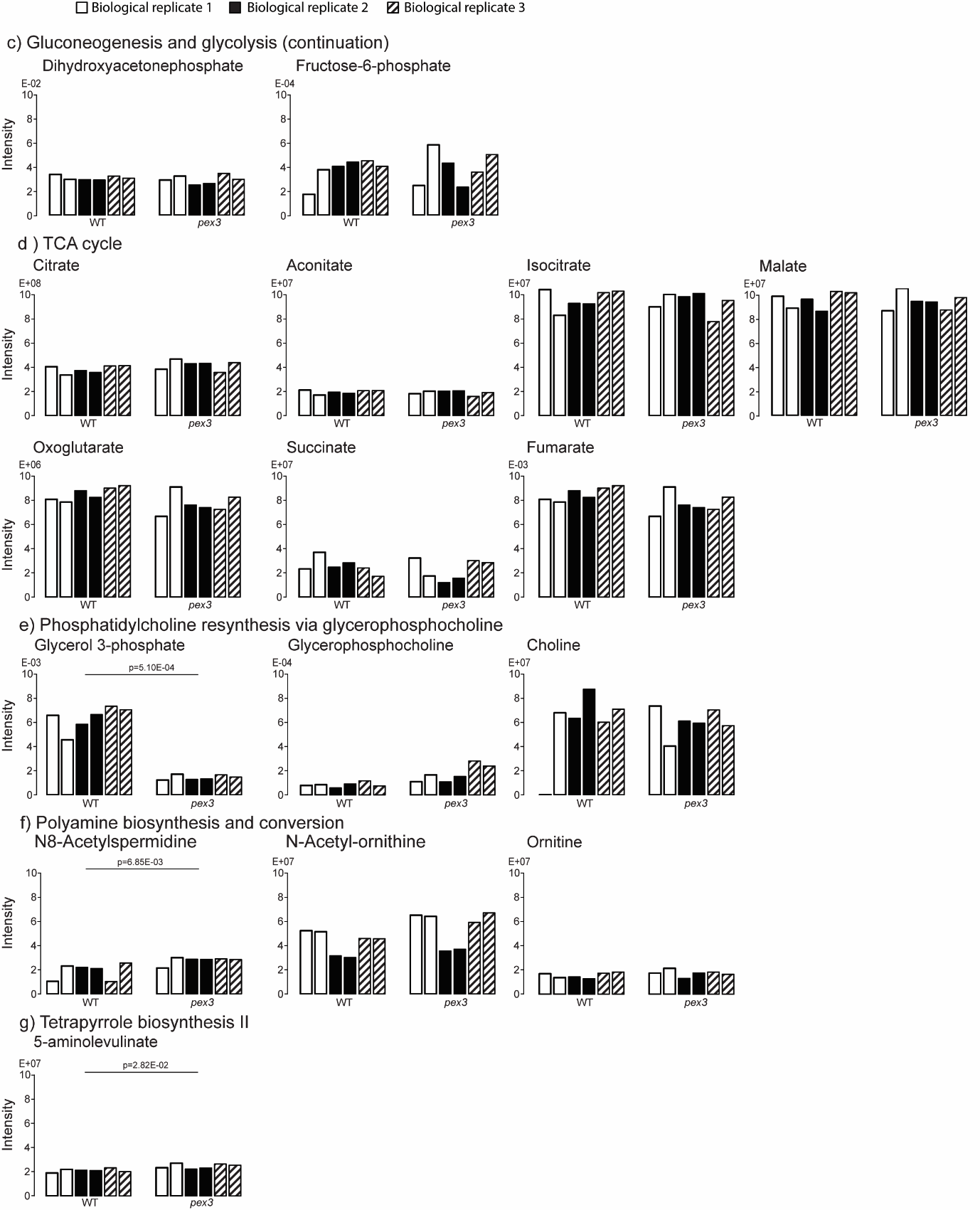


**Supplementary Fig. 1: Results of quantitative metabolomics analysis of *S. cerevisiae* WT and *pex3* cells.** Histograms of the measurements of the quantitative metabolomics analysis of *S. cerevisiae* WT and *pex3* cells of technical duplicates of three biological replicates. Compounds of interest: (a) carnitine related compounds, (b) amino acids, (c) gluconeogenesis and glycolysis, (d) TCA cycle, (e) phosphatidylcholine resynthesis via glycerophosphocholine, (f) polyamine biosynthesis and conversion and (g) tetrapyrrole biosynthesis II. Metabolites that are significantly changed have indicated p-values above the measurements (with addition of lysine).


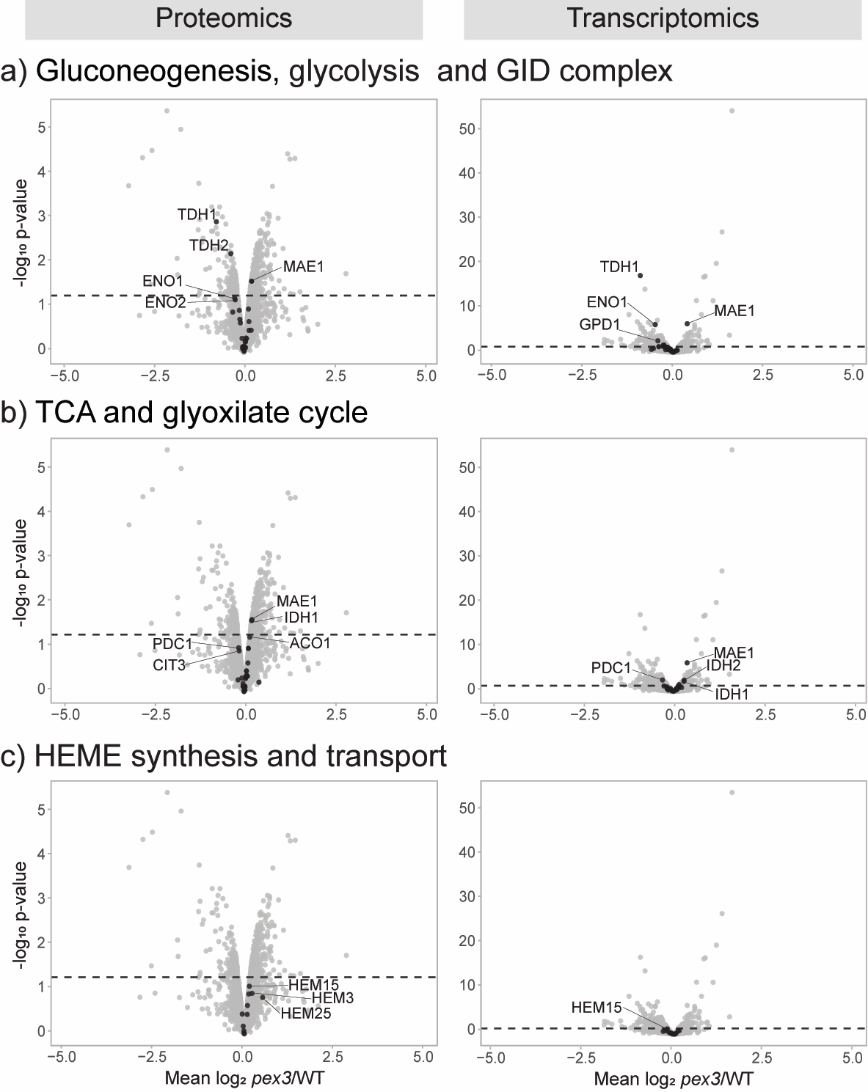


**Supplementary Fig. 2: Quantitative proteomics and transcriptomics analysis of *S. cerevisiae* WT and *pex3* cells.** Volcano plots depicting the results of the quantitative proteomics (left) and transcriptomics (right) analyses of *pex3* versus WT cells highlighting the transcripts and proteins of: (a) gluconeogenesis, glycolysis and GID complex, (b) TCA and glyoxylate cycle and (c) HEME synthesis and transport. The horizontal line in the volcano plots indicates a p-value of 0.05.

## Supplementary Tables

**Supplementary Table 1: Results of quantitative metabolomics analysis of *S. cerevisiae* WT and *pex3* cells** (xlsx file). (a) Intracellular metabolites of *S. cerevisiae* WT and *pex3* cells were analyzed by quantitative mass spectrometry, using biological triplicates and technical duplicates. (b) List of highly annotated metabolites identified with KEGG (confidence level 1 and level 2). Metabolites are categorized into the pathways outlined in the manuscript. (c) Pathway analysis of the highly annotated metabolites using the MetaboAnalyst.

**Supplementary Table 2: Quantitative proteomics and transcriptomics analysis of *S. cerevisiae* WT and *pex3* cells** (xlsx file)**.** Transcriptomics and proteomics candidates of *S. cerevisiae* WT and *pex3* cells (Kosir et al. 2025) with p-value threshold ≤ 0.05 and log2 fold-change of ≤ -0.6 or ≥ 0.59 and ≤ -0.32 or ≥ 0.32, respectively, were considered changed and categorized into the pathways outlined in the manuscript.

**Supplementary Table 3: Yeast strains used in this study.**

| Strain | Description and genotype | Reference |
| --- | --- | --- |
| *Sc* BY4741 (WT) | MATa his3Δ1 leu2Δ0 met15Δ0 ura3Δ0 | Euroscarf #Y00000 |
| *Sc* BY4741 *pex3* | MATa his3Δ1 leu2Δ0 met15Δ0 ura3Δ0 pex3(YDR329c)::kanMX4 | Euroscarf #Y03688 |
| *Sc* WT P*_NOP1_* sfGFP-Fms1 | MATa can1∆::GAL1pr-SceI::STE2pr-SpHIS5 his3∆1 leu2∆0 met15∆0 ura3∆0 hph∆n::URA3::SpNOP1pr-Fms1-Cat2 | (Yofe et al. 2016; Weill et al. 2018) |
| *Sc* WT P*_MDH3_* Fms1-mNeonGreen | MATa can1∆::GAL1pr-SceI::STE2pr-SpHIS5 his3∆1 leu2∆0 met15∆0 ura3∆0 lys2+/lys+ lyp1∆::STE3pr-LEU2 Fms1-mNeonGreen-ADH1term:Hygro | (Meurer et al. 2018) |
